# Oxidative Stress and Lung Ischemia-Reperfusion Injury

**DOI:** 10.1155/2015/590987

**Published:** 2015-06-16

**Authors:** Renata Salatti Ferrari, Cristiano Feijó Andrade

**Affiliations:** ^1^Thoracic Surgery Department, Laboratory of Airways and Lung, Hospital of Clínicas of Porto Alegre, Ramiro Barcelos 2.350, 90035-903 Porto Alegre, RS, Brazil; ^2^Federal University of Rio Grande of Sul (FRGS), Rua Ramiro Barcelos 2400, 2° andar Bairro Santana, 90035-003 Porto Alegre, RS, Brazil

## Abstract

Ischemia-reperfusion (IR) injury is directly related to the formation of reactive oxygen species (ROS), endothelial cell injury, increased vascular permeability, and the activation of neutrophils and platelets, cytokines, and the complement system. Several studies have confirmed the destructiveness of the toxic oxygen metabolites produced and their role in the pathophysiology of different processes, such as oxygen poisoning, inflammation, and ischemic injury. Due to the different degrees of tissue damage resulting from the process of ischemia and subsequent reperfusion, several studies in animal models have focused on the prevention of IR injury and methods of lung protection. Lung IR injury has clinical relevance in the setting of lung transplantation and cardiopulmonary bypass, for which the consequences of IR injury may be devastating in critically ill patients.

## 1. Introduction

The process of ischemia and subsequent reperfusion is present in many medical situations such as major surgical procedures and organ transplantation. This event may lead to devastating consequences in some patients; therefore, the understanding of this process is extremely important in the search for new therapies and procedures that could reduce tissue injury [1].

Tissue damage to a particular organ when subjected to ischemia is exacerbated at the moment of its reoxygenation during reperfusion, a process that is considered to be more harmful than ischemia itself [2]. This mechanism of tissue injury is called reperfusion injury or ischemia-reperfusion (IR) injury and consists of a complex pathophysiological phenomenon requiring the presence of oxygen for its genesis, as well as the maintenance and activation of vascular, humoral, and cellular factors.

In its classical manifestation, occlusion of the arterial supply is caused by an embolus or a plug, resulting in ischemia and consequently a serious imbalance between the supply and metabolic demand, causing tissue hypoxia. During reperfusion, the restoration of blood flow is often associated with an exacerbation of tissue injury and an intense inflammatory response [3].

Ischemia directly affects cells and triggers a series of events due to a lack of oxygen, resulting in different intensities of cellular damage and the consequent activation of cytotoxic enzymes, ultimately culminating in cell death.

Oxidative phosphorylation does not occur in mitochondria during oxygen deprivation; anaerobic glycolysis then begins to provide energy but is not suitable for the replenishment of adenosine triphosphate (ATP). This ATP deficit affects the active transport of ions across the membrane, leading to an accumulation of sodium and, by diffusion, water inside the cell, with subsequent edema. This imbalance also occurs within organelles, leading to the swelling and disintegration of mitochondria and the expansion and formation of vesicles in the endoplasmic reticulum. The rupture of lysosomes and release of enzymes contained therein represent the final events prior to cell death [4].

Reperfusion injury is directly related to the formation of reactive oxygen species (ROS), endothelial cell injury, increased vascular permeability, and the activation of neutrophils and platelets, cytokines, and the complement system [5].

When exposed to hypoxia, endothelial cells alter their cytoskeletal morphology, forming small intercellular pores, and the presence of these pores provides increased permeability of the endothelium, with the formation of tissue edema [6]. The worsening of perfusion is enhanced by an imbalance in the production of vasoconstrictor and vasodilator factors. Hypoxic endothelium shows increased production of potent vasoconstrictors (endothelin types 1, 2, and 3) and decreased production of vasodilators (nitric oxide) [2]. These changes initiated during ischemia, particularly in endothelial cells and leukocytes, not only cause tissue injury but also create conditions that favor future injury with the occurrence of reperfusion. Another effect that has been demonstrated after a period of ischemia reperfusion is the impairment of certain segments of the microcirculation, generating heterogeneity in the distribution of blood flow, with focal tissue hypoxia. This phenomenon, called nonreperfusion (no-reflow), is another mechanism of tissue injury after reperfusion [7].

Due to the complications of IR-induced injury, as well as its high morbidity and mortality, several studies have investigated the pathophysiology of IR injury in an attempt to prevent or reverse its deleterious effects.

## 2. Oxidative Stress and Ischemia-Reperfusion

Oxidative stress has a role in the pathogenesis of several clinical conditions, such as malignancy, diabetes mellitus, atherosclerosis, chronic inflammation, infection with the human immunodeficiency virus, and IR injury [8]. There are different pathways for the production of reactive oxygen species [9], especially via xanthine oxidase as the primary source of production in most organs with systemic vasculature [10]. ROS formation occurs in the mitochondrial matrix through the electron transport chain due to the reduction of molecular oxygen to superoxide radical (O_2_^−^) [11].

When a tissue is subjected to ischemia, a sequence of chemical reactions is initiated. Despite the lack of identification of a critical event responsible for tissue damage, most studies have shown that the depletion of energy and the accumulation of toxic oxygen metabolites (oxidative stress) can contribute to cell death. Paradoxically, reperfusion quickly restores the energy supply by removing toxic metabolites and preventing organ failure; however, it also contributes to and amplifies the mechanisms involved in ischemic tissue damage [5].

During tissue ischemia, a reduction in the availability of ATP as a result of the degradation on adenosine diphosphate (ADP), adenosine monophosphate [12], adenosine, inosine, and hypoxanthine occurs. Furthermore, xanthine dehydrogenase is converted to xanthine oxidase.

This reaction can occur through two mechanisms: (1) xanthine dehydrogenase can be reversibly converted to xanthine oxidase via the oxidation of sulfhydryl groups; or (2) xanthine dehydrogenase can be irreversibly converted to xanthine oxidase via proteolysis through proteases activated by calcium, which is increased in the cytosol and derived from the extracellular environment [5].

Xanthine oxidase relies on oxygen to metabolize hypoxanthine, and when this is provided by reperfusion (reoxygenation), ROS molecules are formed, with a large capacity to cause injury to tissue [5].

**Figure 1:**
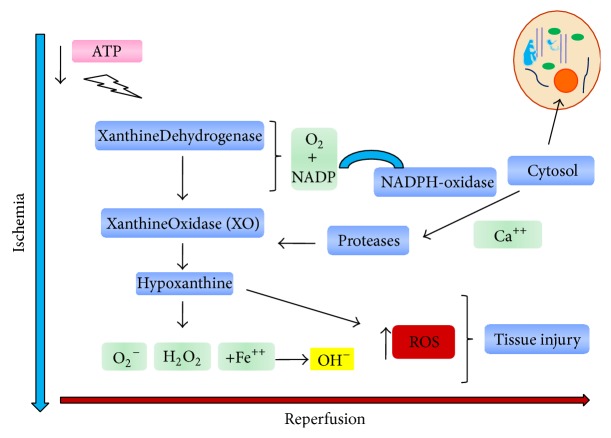
Formation of oxygen-free radicals during lung ischemia and reperfusion injury.

NADPH oxidase, an enzyme expressed in virtually all inflammatory cells, contributes to the formation of cytotoxic peroxynitrite. Furthermore, hydrogen peroxide (H_2_O_2_) derived from the dismutation of O_2_ results in highly toxichydroxyl radical (OH^−^) by the Haber-Weiss reaction, which is facilitated by the increased availability of free iron during ischemia [13] (Figure 1).

Xanthine dehydrogenase uses nicotinamide dinucleotide phosphate (NADP) and may be irreversibly converted to xanthine oxidase. Additionally, proteases are activated by calcium, which are increased in the cytosol. In the presence of oxygen resulting from reperfusion, xanthine oxidase (XO) metabolizes hypoxanthine, forming ROS. Hydrogen peroxide (H_2_O_2_) generates hydroxyl radical (OH), which is highly toxic, by the Haber-Weiss reaction, which is facilitated by the increased availability of free iron during ischemia. The increase in ROS results in major pulmonary tissue damage.

The importance of oxygen radicals in the pathophysiology of IR injury was demonstrated after the injection of free radical scavengers or enzymes, such as superoxide dismutase (SOD), catalase (CAT), and glutathione peroxidase (GPX), preventing the damage that occurs during reperfusion [14, 15].

Several studies have confirmed the destructiveness of the derived toxic oxygen metabolites and their role in the pathophysiology of different processes, such as oxygen poisoning, inflammation, and ischemic injury [16].

## 3. Oxidative Stress and Lung Ischemia-Reperfusion Injury

The mechanisms of IR injury in the pulmonary parenchyma are similar to reperfusion injury in other organs and include a significant involvement of ROS, intracellular calcium influx, endothelial cell injury, leukocyte sequestration and activation in the pulmonary circulation, activation of the complement system, and the release of inflammatory mediators such as arachidonic acid metabolites [2].

Pulmonary IR injury can occur due to trauma, atherosclerosis, pulmonary embolism, and surgical procedures, such as cardiopulmonary bypass (CPB) and lung transplantation [17]. The latter is the most studied situation because it is directly related to the incidence of early graft dysfunction and is responsible for up to 20% of mortality in the early postoperative period [18, 19].

The IR-induced lung injury that occurs in the setting of lung transplantation is characterized by edema, hypoxemia, and pulmonary infiltrates on chest X-ray [20].

This occurs mainly in postcapillary venules, increasing hydrostatic pressure, and favoring the formation of edema, which is facilitated by the increased capillary permeability caused by endothelial injury. ROS have a key role in the development of pulmonary injury (IR) [21, 22], which is characterized by increases in ROS and other free radicals, with a crucial role in the sequence of events leading to lung failure [23].

The IR phenomenon occurs in the heart, liver, kidney, gut, central nervous system, skeletal muscles, and other organs [8]. In these organs, ischemia is accompanied by tissue anoxia until the reintroduction of oxygen during reperfusion and is thus the equivalent to IR anoxia-reoxygenation. Unlike other organs, the lung is considered the only organ that can suffer ischemia without hypoxia because alveolar oxygen helps to maintain aerobic metabolism, thereby preventing hypoxia. Thus, the oxidative stress in the lung resulting from ischemia should be distinguished from that resulting from hypoxia itself [24].

In the setting of lung transplantation, factors present in the prereperfusion phase of the graft, such as brain death, pneumonia, mechanical ventilation, aspiration, contusion, hypotension, and cold ischemia, have been recognized as aggravating IR injury through the activation of inflammatory factors [24, 25].

Hypoxia and consequently anoxia result in a decrease in intracellular ATP and an increase in ATP degradation products, such as hypoxanthine, which generates ROS production when oxygen is reintroduced during reperfusion and/or ventilation. During ischemia, this phenomenon may occur in the lung if the alveolar oxygen tension drops below 7 mmHg [26, 27]. The absence of pulmonary blood flow leads to lipid peroxidation, even in the presence of oxygen. The mechanism of oxidative stress is different from what occurs during anoxia-reoxygenation because it is not associated with decreased ATP, and it may occur even during the period of cold ischemia in an organ stored for transplantation [28].

In the lungs, ROS are related to the activation of inflammatory processes through transcription factors such as nuclear factor-kappa B (NF-*κ*B), leading to chromatin remodeling and the expression of proinflammatory mediator genes [29, 30]. Intracellular ROS production has been observed in various cell types of lung tissue, including endothelial cells, alveolar type II epithelial cells, clara cells, ciliated epithelial cells, and alveolar macrophages [31]. It is believed that IR pulmonary injury is due to an increase in ROS, which triggers a response from the graft, resulting in the activation of the adaptive immune response (acute rejection) through the activation of antigen-presenting cells [32]. Additionally, the use of LPD (low-potassium dextran), a lung preservation solution, appears to decrease ROS production [33] and reduce the incidence of primary graft failure through a reduction in ROS production from the pulmonary vasculature [34].

## 4. Systemic Effects of Ischemia-Reperfusion Injury

IR injury and multiple organ failure contribute significantly to mortality and postoperative morbidity, and reperfusion induces the oxidative stress that plays a key role in this pathology. Pulmonary IR injury induces systemic effects in the liver and heart and is characterized by neutrophil sequestration and the release of significant amounts of ROS into the circulation [35, 36].

However, the pulmonary system may also suffer consequences from IR tissue located remotely [37]: a single organ exposed to IR can subsequently cause inflammatory activation in other organs, leading to the failure on multiple systems. Importantly, ischemic syndromes are a heterogeneous group of conditions. Although there are some similarities in biological responses between these syndromes that occur in different organs, there are important differences between a reduction in systemic perfusion, for example, during shock, compared with regional ischemia and the reperfusion of a single organ [38].

During IR injury in the liver or kidney, the activation of intestinal inflammatory responses triggers a sequence of events that leads to multiorgan failure. The IR of peripheral organs (such as the liver) results in the activation of intestinal Paneth cells and the subsequent release of cytokines such as IL-17, causing failure in other systems, including the pulmonary system [39, 40].

The systemic inflammatory responses of mesenteric IR represent an important model of severe disease because deficits in the intestinal mucosa appear to be critical in the initiation and propagation of multiple organ failure [41].

Using a mouse model of intestinal IR injury, Mura et al. reported that nearly 50% of the IR group animals died during the experimental period of 4 h. The combined effects of intestinal IR, surgical procedure, the application of a high oxygen concentration, and mechanical ventilation may be responsible for this high mortality rate. In this model, the lung was the most severely injured remote organ [4]. Additionally, a recent clinical study confirmed that respiratory dysfunction following traumatic injury is an obligatory event that precedes heart, kidney, and liver failure [4, 42].

Recent studies also report that activated neutrophils aggregate in the subendothelial space, where they release reactive oxygen species (ROS), enzymes, and cytokines, causing direct renal injury and the recruitment of monocytes and macrophages leading to further aggravation of the oxidative injury [43, 44].

## 5. The Antioxidant Defense System 

An antioxidant is any substance that, even at low concentrations, significantly delays or inhibits the oxidation of a substrate in an enzymatic or nonenzymatic manner [45].

The organism's defense against ROS ranges from the prevention of ROS formation to interception of the formed radicals to cell repair. Enzymes that control the levels of ROS are glutathione peroxidase (GPx), superoxide dismutase (SOD), and catalase (CAT), leading to the sequestration and deactivation of ROS, which are neutralized to prevent the further oxidation of other molecules. The final neutralization of a compound with one or more unpaired electrons is the formation of another nonradical product.

Water-soluble radical compounds transfer the radical function away from the potential target site and are called free radical scavengers. The combination of a substance with a free radical leads to the formation of a nonradical or a radical that is less harmful, for example, tocopherols and carotenoids [45].

Antioxidant therapy can be performed by the replacement of endogenous antioxidants, such as recombinant superoxide dismutase [46] or by exogenous supplementation of antioxidant agents, such as N-acetylcysteine [47].

However, the use of antioxidants in animal models of lung injury has been little exploited in experimental and clinical studies, for example, the use of N-acetylcysteine [48], which has proven to be an important therapeutic potential for use in IR lesion [42].

## 6. N-Acetylcysteine

Several authors have described different ways to increase the viability of lung graft posttransplantation and to reduce the undesirable effects of IR injury, including the use of antioxidants such as NAC and melatonin [42, 49].

NAC (chemical formula C_5_H_9_NO_3_S; molecular weight 163.2) is a thiol compound that contains a sulfhydryl group and is widely used in clinical medicine [50].

NAC is a mucolytic that was first implemented for treating congestive and obstructive lung diseases associated with hypersecretion. NAC is also used in the treatment of adult respiratory distress syndrome and in cases of acquired immunodeficiency in HIV infection [51]. Its antioxidant activity is mainly governed by two mechanisms: (1) direct reduction of H_2_O_2_ and O_2_^•−^ into less reactive species, forming sulfur or cysteine radicals; (2) promotion of the biosynthesis of GSH, which acts as a scavenger of free radicals and as a substrate in the redox cycle of glutathione [52].

The loss of antioxidant capacity in an oxidized cell is mainly due to a decrease in glutathione, which is the most abundant intracellular free thiol. Oxidative stress in vivo is translated as a deficiency in glutathione or its precursor, cysteine, and the most effective antioxidant that has been studied is the NAC, a glutathione precursor [46].

Chemically, NAC is similar to cysteine, and the presence of this acetyl environment reduces thiol reactivity compared to cysteine. Moreover, NAC is less toxic and less susceptible to oxidation and dimerization and is more soluble in water, making it a better source of cysteine than the parenteral administration of cysteine [53]. NAC provides protection mediated by administration of lipopolysaccharide endotoxemia, resulting in a decrease in H_2_O_2_, and this was directly related to its ability to reduce ROS rather than its function of promoting the biosynthesis of glutathione [54]. Many studies have demonstrated the effects of NAC, mainly with regard to modulating the activity of inducible nitric oxide synthase (iNOS), reducing the formation of inflammatory cytokines and inhibiting the action of neutrophils [55–58]. Furthermore, NAC acts as a “scavenger” of free radicals by inhibiting oxidative stress and preventing cell death [59].

Studies have demonstrated that treatment with NAC prior to lung warm ischemia significantly attenuates inflammatory changes in both the ischemic and reperfusion periods [60]. NAC reduces the phosphorylation of I*κ*B-*α* and p-65, resulting in a decrease in apoptosis and inflammatory responses. The intravenous administration of NAC demonstrates protective properties against lung IR injury, and the use of NAC immediately after reperfusion potentiates its protective effects [61].

Study of Wu et al., demonstrated that the NAC administration reduced lung I/R-induced increases in myocardial hydroxyl radical production and lipid peroxidation and ameliorated LV contractility and stiffening [62].

This protective effect could be explained by NAC increasing GSH synthesis or eliminating free radicals directly or both. The observed reduction in malondialdehyde (MDA) levels is consistent with the potent antioxidant effects of NAC, with a significant reduction in lipid peroxidation being reported by many researchers [63–65].

Current studies suggest the use of isoprostane as a more specific index of oxidative stress induced by ROS [66–68].

In experimental studies, treatment with NAC resulted in higher levels of tissue GSH, which led to improved lung graft function [46, 69].

## 7. Calcium and Sodium Pump in Ischemia-Reperfusion Injury

Ischemia causes an increase in calcium permeability by promoting its entry into cells. Such an increase in intracellular calcium, which is enhanced by a decrease in its active, ATP-dependent transport to the extracellular environment, has several deleterious effects: a change in cell shape by contraction of the cytoskeleton and phospholipase activation, with the consequent release of the metabolite arachidonic acid from cell membranes and organelles and the production of free radicals. All these effects contribute to cell death [70].

In addition to the increase in intracellular calcium as a result of ischemia, intracellular consumption occurs during the “storage” of ATP; hence, there is an increase in anaerobic glycolysis products. Such an event impairs the transmembrane ion gradient, with the consequent accumulation of sodium (Na^+^) and water, leading to cellular edema and the swelling of organelles, such as the mitochondria, culminating in cell lysis. Furthermore, the sodium pump (Na^+^/K^+^ATPase) is inactive during ischemia, contributing to the disruption of the ion gradient [71].

The accumulation of calcium ions (Ca^++^) intracellularly as a consequence of changes in the permeability of the plasma membrane and the decrease in its active ATP-dependent transport results in the activation of phospholipases and proteases [72]. Proteases potentiate the effects of ROS on organelles by converting xanthine dehydrogenase to xanthine oxidase, and phospholipases activate the transformation of arachidonic acid into products such as leukotrienes, prostaglandins, and thromboxane [72].

## 8. Endothelium in Lung IR Injury

The endothelium is the main source of ROS during nonhypoxic pulmonary ischemia through the activation of NADPH oxidase. This enzyme complex is also found in other lung cells, but its concentration is more evident in neutrophils, monocytes, and alveolar macrophages. Cell stimulation during ischemia results in the translocation of NADPH oxidase components to the cell membrane, a site where integration occurs with membrane components to form a system of electron transfer that catalyzes the reduction of molecular oxygen (O_2_) to superoxide radical (O_2_^−^) while oxidizing NADPH. This increase in the consumption of O_2_ and production of O_2_^−^ is responsible for the “oxidative burst” that results from the activation of NADPH. The O_2_^−^ generated can be subsequently transformed into H_2_O_2_ in a reaction catalyzed by SOD. Oxidizing compounds are also produced by enzymes contained in intracellular granules. Azurophilic granules release the enzyme myeloperoxidase [12], which during neutrophil activation catalyzes the reaction between H_2_O_2_ and chlorine to produce hydrochloridric acid, which is considered to be an extremely potent oxidant. Furthermore, hydrochloridric acid can react with amines, generating chloramines, which are considered potent oxidants [73].

Based on previous studies that demonstrated the presence of the enzyme xanthine dehydrogenase in endothelial cells and the ability of the cells to release ROS, these authors studied the effect of the presence of activated neutrophils in contact with these cells. They demonstrated that activated neutrophils induce the conversion of xanthine dehydrogenase into xanthine oxidase in endothelium [74].

## 9. Iron 

Although iron is an essential element for all cells, it may be highly toxic under pathophysiological conditions, such as in the presence of oxidative stress, due to its oxidation-reduction properties [41].

The iron is mostly stored in ferritin molecules and is transported by transferrin molecules. However, “free” iron exists and can participate in Fenton's reaction, in which O_2_ and H_2_O_2_ react with iron and produce OH radicals. Iron is released from ferritin and cytochrome P-450 during ischemia due to the effect of acidosis and through the action of superoxide radical and proteolysis. Moreover, when released into the circulation, iron can activate platelet aggregation. An experimental model of lung IR injury using 3 hours of warm ischemia in dogs showed that a new type of lazaroid is able to reduce iron-dependent lipid peroxidation [75].

## 10. Inflammatory Mediators 

Some inflammatory mediators released as a consequence of the reperfusion of an organ or similar such region can activate endothelial cells in distant organs that were not exposed to the ischemic insult but are injured as a result of reperfusion injury.

Moreover, reperfusion injury is characterized by autoimmune responses, including the recognition of natural antibodies and neoantigens and the subsequent activation of the complement (autoimmunity) system [76]. Despite the fact that IR typically occurs in a sterile environment, the activation of innate and adaptive immune response occurs and contributes to the injury, including the activation of pattern recognition molecules, such as toll-like receptors (TLR), and the inflow of inflammatory cells in the injured organ [77].

**Figure 2:**
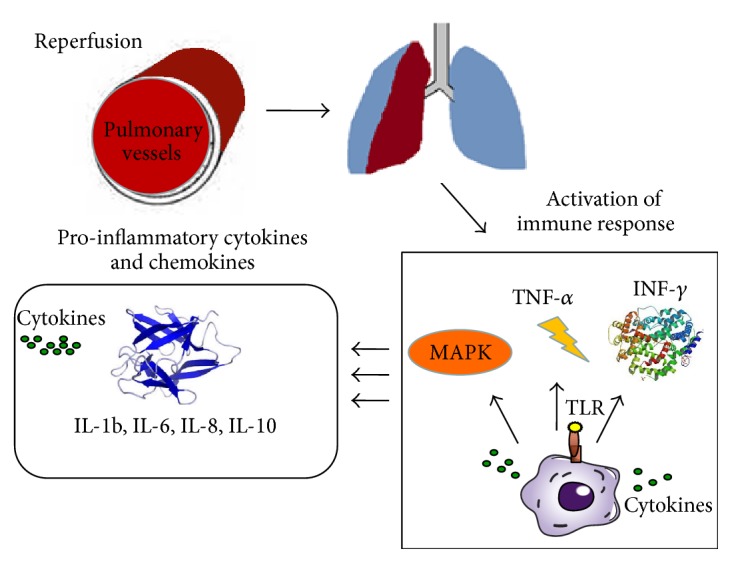
Activation of the immune response and trafficking of inflammatory cells in the diseased organ during reperfusion.

For example, when TLRs recognize specific molecules, they trigger the activation of signaling pathways, including the NF-*κ*B activation of protein kinase (MAPK) pathways and type I interferon, which results in the induction of proinflammatory cytokines and chemokines. These receptors can also be activated by endogenous molecules in the absence of microbial compounds, particularly within the context of cell damage or death, as occurs during IR [78] (Figure 2).

The recognition of “danger signals” by toll-like receptors (TLRs) on the surface of inflammatory cells leads to the activation of different signaling pathways, including the NF-*κ*B activation of protein kinase (MAPK) pathways and type I interferon, which results in the induction of proinflammatory cytokines and chemokines.

Specifically, the activation of TLR4 may be aggravated by the oxidative stress generated during IR, which when recognized by inflammatory cells increases responsiveness to subsequent stimuli. Alveolar macrophages from rodents subjected to hemorrhagic shock and resuscitation express increased levels of TLR4, an effect that was inhibited by the addition of the antioxidant NAC with fluid resuscitation [77]. Andrade et al. examined the levels of TLR mRNA expression in lung tissue collected during IR in human lung transplantation and found that the mRNA levels of most TLRs correlate with the mRNA levels of cytokines (IL-1b, IL-6, IL-8, IL-10, and IFN-gamma) in the lungs of donors during hypothermic storage. These observations suggest that inflammatory responses in the donor organ can affect the expression and activity of TLR genes; alternatively, the levels of expression and activation of TLRs may contribute to the regulation of cytokine gene expression. In addition, a close correlation between TL4 and IL-8 before and after reperfusion was found, suggesting that this cytokine may be involved in the regulation of TLR4 gene expression in the setting of lung transplantation [79].

TNF-*α* (tumor necrosis factor *α*), ROS, and interleukin-6 (IL-6) are involved in the tissue damage that occurs during IR because they are toxic molecules that alter cellular proteins, lipids and ribonucleic acids, leading to cellular dysfunction or death [80]. A further contribution to tissue injury occurs when the worsening of perfusion is potentiated by an imbalance in the production of vasoconstrictor and vasodilator factors. The hypoxic endothelium shows an increased production of potent vasoconstrictors (endothelin types 1, 2, and 3) and a decreased production of vasodilators (nitric oxide) [2].

The cellular damage generated by ROS in the lipid membrane promotes the activation of phospholipase A2 by inducing the production of platelet activating factor (PAF), which promotes the mobilization of arachidonic acid from the phospholipids of cell membrane. Arachidonic acid is the substrate for numerous enzymes and inside the lungs is primarily metabolized by two enzymes, cyclooxygenase and 5-lipoxygenase, producing inflammatory mediators. The cyclooxygenase pathway generates prostaglandins (PGE1 and PGI2) and thromboxane (TXA2), and the 5-lipoxygenase pathway produces leukotrienes, such as leukotriene B4, C4, D4, and E4 [81, 82].

Pulmonary vascular resistance depends on the interaction between vasoconstrictor and vasodilator factors. Most of the metabolites of arachidonic acid are derived from endothelial cells and contribute to maintaining low vascular resistance in the lung. The effects of prostaglandins and thromboxanes are antagonistic. Prostacyclin (PGI2) is a bronchodilator and a pulmonary vasodilator and prevents platelet aggregation, whereas thromboxane A2 (TXA2) is a broncho- and vasoconstrictor and induces platelet aggregation [82].

Prostaglandins (PGE1 and PGI2) are associated with the following effects: vasodilation and bronchodilation; the inhibition of platelet aggregation, leukocyte adhesion, and sequestration; and the suppression of proinflammatory cytokine (TNF-*α*, IL-1, and IL-6) production [12, 83, 84].

PAF can be released from various cells, such as macrophages, platelets, mast cells, endothelial cells, and neutrophils, and is responsible for leukocyte activation, platelet aggregation, cytokine release, and adhesion molecule expression [85]. PAF acetylhydrolase is responsible for the degradation and regulation of the activity of PAF, and high levels of this enzyme were found in the bronchoalveolar lavage of patients with ARDS [86]. Furthermore, it has been observed that when added to a lung preservation solution in an isolated perfused rat model, the substance has the ability to reduce pulmonary capillary permeability [87].

Leukotrienes, products of arachidonic acid metabolism by the 5-lipoxygenase pathway, are divided into two classes: cysteine LTC4, LTD4, and (LTE4) and noncysteine (LTB4). LTB4, a potent proinflammatory activator of leukocyte chemotaxis that has an important role in lung IR injury, is produced by monocytes, lymphocytes, mast cells, and lung macrophages [88].

Vascular endothelial growth factor (VEGF) and its receptor are central to the regulation of vascular permeability and the survival of endothelial cells. Mura et al. suggested that VEGF may have dual roles in LPA-induced intestinal IR. The early release of VEGF can increase pulmonary permeability, whereas a decrease in the expression of VEGF and VEGFR-1 in lung tissue could contribute to the death of alveolar epithelial [4].

## 11. Nitric Oxide 

Nitric oxide (NO) plays an important role in vascular homeostasis due to its potent vasoregulatory and immunomodulatory properties. It is known that NO attenuates the capillary overflow and tissue damage observed in animal models of pulmonary IR, myocardial and cerebral ischemia by inhibiting the adhesion of neutrophils, and the production of superoxide by neutrophils [89].

NO is considered to be an optimal transcellular messenger due to its lipophilic nature and short half-life in biological systems, approximately 3 to 30 seconds [90].

NO is also a key biological mediator produced by various cell types, including vascular endothelium, is an inhibitor of platelet aggregation and neutrophil adhesion, and modulates vascular permeability. Additionally, NO acts as a bronchodilator and neurotransmitter [84].

After pulmonary IR, the levels of endogenous NO are reduced. This may be associated with the increased expression of eNOS, which may suggest that endogenous NO production can be readily attenuated by free radicals after reperfusion and/or because IR can induce the generation of NOS inhibitors [91, 92].

The decreased production of endogenous NO by the immediate reaction of NO with the radical superoxide results in the production of a powerful oxidant, peroxynitrite (OONO). Such a loss of the protective action of NO will result in endothelial dysfunction [90].

## 12. Leukocyte Activation

IR injury in lung transplantation has a biphasic pattern. The early phase of reperfusion is mainly dependent on the characteristics of the donor, whereas the late phase is dependent on the characteristics of the recipient, lasting 24 hours. Donor macrophages activated during ischemia are the mediators of the early phase; lymphocytes and neutrophils from the recipient are mainly involved in the late phase. The recruitment of these cells occurs through the release of cytokines and other inflammatory mediators before and after reperfusion [93].

Alveolar macrophages produce large amounts of cytokines and procoagulant factors in response to oxidative stress. The importance of tumor necrosis factor alpha (TNF-*α*), interferon gamma (INF-*γ*), and chemoattractant protein-1 macrophage (equivalent to human IL-8) in the early phase of graft reperfusion was shown in a rat lung IR model [94].

Lymphocytes play an important role in pulmonary IR injury. The lung contains a large amount of donor macrophages and activated lymphocytes represented by T and natural killer cells, which are responsible for the graft-host immune response but also have beneficial immunomodulatory effects [95]. In a rat lung transplant model, it was observed that CD4+ T lymphocytes are the mediators of IR injury and infiltrate the graft an hour after reperfusion, consequently increasing the production of IFN-*γ*; furthermore, it was suggested that this effect is independent of neutrophil recruitment and activation [24].

The role of leukocytes in lung reperfusion injury is due to the release of substances during their own degranulation, some of which are free radicals. Polymorphonuclear neutrophils possess nicotinamide adenine dinucleotide phosphate oxidase (NADPH oxidase), which is capable of reducing molecular oxygen and generating superoxide anion [96].

These cells also secrete myeloperoxidase enzyme, which catalyzes the formation of hypochlorous acid (HOCl) from the oxidation of chloride ion in the presence of hydrogen peroxide. HOCl reacts with amines, generating chloramines, potent oxidants [97].

Neutrophils have the characteristic of infiltrating the transplanted lung progressively during the first 24 hours after reperfusion. Although these cells have an important role in the late phase of reperfusion injury, their role in the early phase is less well known. Deeb et al. demonstrated that reperfusion injury in the first four hours depends of the presence of neutrophils, with macrophages also having an important role at this stage; none the less, after this period, neutrophils are the primary mediators [98].

## 13. Apoptosis and Lung Ischemia-Reperfusion Injury

Apoptosis is an active process, the hall mark of which is the controlled autodigestion of cellular constituents due to the activation of endogenous proteases and can be metaphorically compared to “cell suicide.” The activation of these proteases compromises the integrity of the cytoskeleton, causing the collapse of the cell structure. In response to the contraction of the cytoplasmic volume, the cell membrane forms bubbles, with changes in the positioning of the lipid components [99].

Nuclear NF-*κ*B transcription is regulated by the inhibitory action of inhibitor of *κ*B (I*κ*B), which is targeted for degradation via phosphorylation by the action of I*κ*B kinases (IKK*α*, IKK*β*) [100]. Inflammatory signaling activates a cascade of events, such as the phosphorylation of the TNF receptor, leading to the activation of transforming-growth factor b-activated kinase 1 (TAK1). TAK1 phosphorylates the IKK complex and then phosphorylates I*κ*B*α*, resulting in the ubiquitination and dissociation of I*κ*B*α* from NF-*κ*B and I*κ*B*α* degradation by the proteasome. NF-*κ*B translocates to the nucleus and binds to specific DNA regions, initiating the transcription of multiple genes, including cytokines, chemokines, and other inflammatory mediators [101].

**Figure 3:**
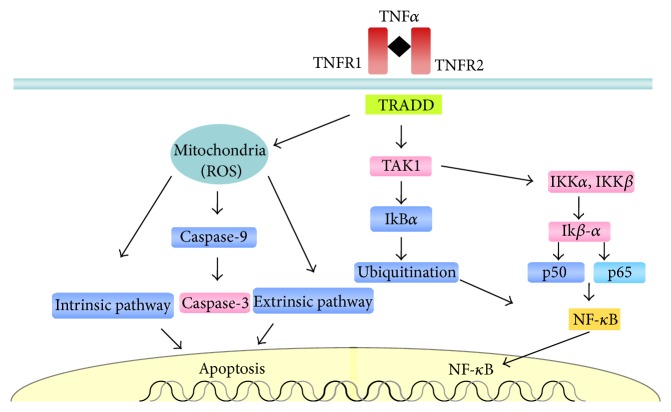
The transcription of nuclear NF-*κ*B is regulated by the inhibitory action of the inhibitor protein I*κ*B during ischemia-reperfusion injury.

Unlike what occurs with necrosis, apoptosis or programmed cell death does not occur during ischemia, but a peak of it does occur during reperfusion [100]. The induction of apoptosis can occur through two pathways. The intrinsic pathway is dependent on mitochondria and is activated by ROS, whereas the extrinsic pathway is dependent on inflammatory molecules, such as TNF-*α*. However, by activating the production of ROS via the NADPH oxidase pathway, TNF would also contribute to the intrinsic pathway [102]. Both pathways promote the activation of caspases and proteases responsible for the cleavage of specific cellular substrates, which results in changes in cellular configuration, changes in membrane permeability, and DNA fragmentation, with consequent cell death [103]. The intrinsic pathway is activated in the early phase of reperfusion, and the extrinsic pathway can be activated up to a few hours after reperfusion [104] (Figure 3).

Phosphorylation of the TNF receptor (TNFR1 and TNFR2) leads to the activation of transforming-growth factor b-activated kinase 1 (TAK1), which phosphorylates the protein I*κ*B*α*, resulting in ubiquitination and leading to the dissociation of I*κ*B*α* from NF-*κ*B. TAK1 also leads to degradation of IKKa and IKKb, releasing two subunits of p50 and p65; NF-*κ*B translocates to the nucleus, initiating the transcription of multiple genes, including cytokines, chemokines, and other inflammatory mediators. This occurs to be concomitant with the induction of apoptosis by the activation and induction of two pathways: the mitochondria-dependent intrinsic pathway is activated by ROS, and the extrinsic pathway is dependent on inflammatory molecules, such as TNF-*α*. The intrinsic pathway is activated in the early phase of reperfusion; the extrinsic pathway can be activated up to a few hours after pulmonary IR.

Apoptosis is regulated by a cascade of proteins called caspases, which are apoptosis effector proteins present in all cells. After cleavage, caspases become active and initiate pathways leading to apoptosis [105].

The following are features of apoptosis: chromatin condensation, phosphatidylserine exposure on the cell surface, cytoplasmic shrinkage, the formation of apoptotic bodies, and fragmentation of DNA [106]. As opposed to necrosis, which also occurs in the absence of ATP, apoptosis is an energy-dependent process [107].

Forgiarini et al. demonstrated that the duration of ischemia has a direct effect on the viability of lung cells using an experimental model of lung IR. The increase in caspase 3 activity reflected a larger number of apoptotic cells after 45 minutes of ischemia [108].

The signaling pathway that leads to programmed cell death is maintained by positive and negative regulators, and the balance between these factors decides whether the cell survives or undergoes apoptosis. The proteins that promote survival are the antiapoptotic proteins Bcl-2 and Bcl-xL, whereas proapoptotic proteins Bax, Bad, Bak, and Bid induce programmed cell death [109].

**Figure 4:**
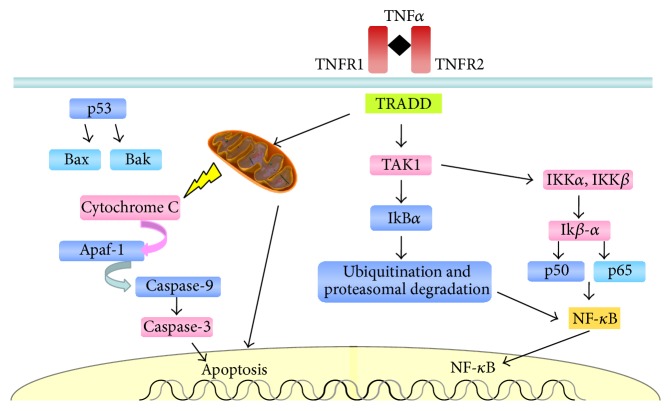
Release of cytochrome C from mitochondria triggers the activation of caspase 9, which cleaves caspases 3 and 6, leading to apoptosis.

An important regulator of apoptosis following DNA damage is p53, which can induce Bax and Bak, regulating the release of cytochrome C from mitochondria and thereby initiating the cascade leading to apoptosis [110]. Cytochrome C binds to apoptotic protease activating factor 1 (Apaf-1), activating caspase 9, which in turn cleaves caspases 3 and 6 [111, 112], leading to cell death (Figure 4).

An important regulator of apoptosis is p53, which can induce Bax and Bak, which regulate the release of cytochrome C from mitochondria, thereby initiating the cascade that leads to apoptosis. Cytochrome C binds to apoptotic protease activating factor 1 (Apaf-1) to activate caspase 9, which cleaves caspases 3 and 6, leading to cell death.

## 14. Prevention and Treatment of Pulmonary IR Injury

Major advances in our understanding of the mechanisms of reperfusion injury and the development of strategies to increase tissue resistance to ischemia or to attenuate reperfusion injury have occurred. For example, experimental studies of adaptive responses induced by hypoxia have provided strong evidence for new treatment approaches in IR [38].

The tissue damage is not limited only to ischemia and may extend or worsen with reperfusion, and recognizing this is important for carefully reversing ischemia, which is a critical point for maintaining tissue viability under damaging conditions [20].

Because of the pulmonary damage that IR causes, many studies in animal models have focused on the prevention of IR injury and the improvement of lung preservation methods [113–115], such as the use of lung hyperinflation [26, 116], hypothermic preservation [117], different lung preservation solutions [118, 119], retrograde pulmonary perfusion [118, 119], liquid ventilation [120], and perfluorocarbon [121, 122]. These are in addition to the use of vasodilators [123, 124] antioxidants [125] gene therapy [126], inhaled nitric oxide [89], and ischemic preconditioning (PCI) [127].

All therapeutic options tested by different methods attempt to minimize or prevent the cell death that occurs during IR and consequently activate the various pathways of cell death, which can be categorized as necrosis, apoptosis, or cell death associated with autophagy. Necrosis is characterized by the swelling of cells and organelles with the subsequent rupture of membranes and the surface and the shedding of intracellular contents [128].

Necrotic cells are highly immunostimulatory and cause inflammatory cell infiltration and cytokine production. In contrast, apoptosis involves a cascade of caspase signaling that induces programmed cell death, which is characterized by cell and nuclear shrinkage, though the integrity of the plasma membrane persists until the end of the process. Different studies have investigated whether the inhibition of apoptosis may become a promising therapeutic strategy for lung ischemia-reperfusion injury [129, 130].

Some studies have investigated whether mice with a disruption in the gene encoding IKKb, the catalytic subunit of IKK that is essential for the activation of NF-*κ*B, can provide an opportunity to study the effects of preventing the activation of NF-*κ*B. However, this manipulation results in embryonic lethality due to massive apoptosis in the developing liver driven by NF-*κ*B [131]. Ishiyama et al. [132] studied the inhibition of NF-*κ*B activation by applying inhibitors that prevented I*κ*B phosphorylation and showed an increase in the oxygenation of the transplanted lung and reductions in pulmonary edema, neutrophil aggregation, and apoptotic cells after experimental lung transplantation.

Chang and Yang [103] demonstrated that the inhibition of NF-*κ*B attenuates IR injury, as it is responsible for a reduction in cytokine production. In their study, the activation of NF-*κ*B was responsible for the increased expression of caspase 3 and iNOS.

Another study on intestinal ischemia and reperfusion showed that although IKKb deficiency in enterocytes is associated with a reduction in inflammation, severe apoptotic damage occurs in the mucosa. Thus, attempts to inhibit the activation the NF-*κ*B pathway are associated with the prevention of systemic injury but consequently increase local inflammation injury [133].

Certain calcium channel blockers are also used, such as verapamil, which has a protective effect during lung ischemia-reperfusion [134].

Torres et al. suggested that the presence of LPD preservation solution in the systemic blood increases the plasma's total antioxidant potential, both in the presence and absence of a lung ischemic event. A decrease in erythrocyte LPD was also observed in the presence of lung ischemia [33].

Other evidence suggests that TLRs are involved in IR injury of different organs. In a study of myocardial ischemia-reperfusion injury, two strains of TLR4-deficient mice (C57/BL10 SCCR and C3H/HeJ) showed significantly smaller areas of myocardial infarction than control strains (C57/BL10 ScSn and C3H/OuJ). The TLR4-deficient mice also showed reduced neutrophil infiltration, reduced lipid peroxidation, and reduced complement deposition in cardiac tissues [120, 135].

Some studies have used NO as an additional substance for lung preservation, and this has been shown to be effective in reducing the damage of reperfusion injury in various animal models [136–139]. However, the use of NO during lung reperfusion did not decrease pulmonary edema in a randomized clinical trial [89]. In another study of 84 patients undergoing lung transplantation, the use of NO during reperfusion showed no benefit with respect to hemodynamics, extubation, the incidence of IR injury, or the length of hospital and ICU stay [140]. Ardehali and colleagues [141] demonstrated a benefit with the use of inhaled NO postoperatively a subgroup of patients who developed IR injury despite not decreasing its incidence [141].

## 15. Conclusion 

Over the years, several studies have investigated possible therapeutic alternatives that are deemed safe and with proven clinical efficacy. Although these alternatives may act directly on tissue damage triggered by ischemia and reperfusion, clear safety and effective evidence have yet been clinically demonstrated.

## Future Perspectives

The search for different methods of lung protection is necessary and indispensable for testing different pharmacological approaches in an attempt to provide better therapeutic strategies for IR injury.
